# NIEHS's New Strategic Plan

**DOI:** 10.1289/ehp.1205642

**Published:** 2012-08-01

**Authors:** Linda S. Birnbaum

**Affiliations:** Director, NIEHS and NTP, National Institutes of Health, Department of Health and Human Services, Research Triangle Park, North Carolina, E-mail: birnbaumls@niehs.nih.gov

I am pleased to present “our” new strategic plan for the National Institute of Environmental Health Sciences (NIEHS 2012). I say that it’s “our” plan because the entire document reflects tremendous thought, discussion, and sharing of ideas by hundreds of scientists and community stakeholders, and it speaks to the entire field of environmental health research. This plan is about what we will strive to accomplish together as we devote ourselves to research that, in my opinion, has the greatest chance for preventing disease and for improving health throughout the world.

As reflected in the new strategic plan (NIEHS 2012), the NIEHS has a fresh vision, not because our values have changed but because our research has been so successful—and many of you have made a huge contribution to that progress.

New technologies and increasing knowledge bring exciting new opportunities each and every year. The NIEHS’s new strategic plan builds upon the accomplishments and vision that came before.

The NIEHS has come a long way in making environmental health research responsive to the needs and concerns of the American people— to make environmental health part of the public health debate. This continues to be a source of motivation and purpose for NIEHS staff and our research partners. Environmental justice is an everlasting core value for NIEHS research.

In the past few years, we at the NIEHS have made some important progress in exposure science, supporting new technologies for sensor devices and bioinformatics. We have been embracing new science such as epigenetics and exposure phenotyping, focusing on interdisciplinary research and translational research. In addition, our clinical research unit is now up and running.

As the NIEHS moves forward, our overall goal is to make the institute, including the National Toxicology Program (NTP), the foremost trusted source of environmental health knowledge, leading the field in innovation and the application of research to solve health problems.

Our new vision statement (NIEHS 2012) captures our collective dreams and aspirations, and reflects our strong commitment to making a real difference:

The vision of the National Institute of Environmental Health Sciences is to provide global leadership for innovative research that improves public health by preventing disease and disability from our environment.

What this means in practical terms is that we are pursuing some of the “big influences” that have been understudied, all of which interact with traditional environmental exposures: the microbiome, for example, and inflammation pathways, immunological pathways, nutrition, and epigenetic processes. We also want to lead the process of defining the “exposome,” which is the totality of exposure encountered by humans.

We have elevated the NTP to the divisional level within the NIEHS. And we will continue to integrate our toxicology research with our excellent basic and translational science programs, not because I am a toxicologist but because the NTP is a problem-solving program—a truly translational component of the NIEHS. For example, the NTP is part of our consortium on bisphenol A, contributing to the scientific deliberations, right along with our intramural scientists and our grantees.

The NTP is leading the Tox21 initiative along with the National Institute of Health’s National Center for Comparative Genomics, the U.S. Environmental Protection Agency, and the Food and Drug Administration. This high throughput testing program shows great promise not only as a new and faster method but also for moving toxicology into a predictive science.

The NTP is moving beyond the traditional approaches of testing one chemical at a time and are taking on the significant challenge of evaluating mixtures. We are also looking at the effects of exposures throughout the life span, expanding our research and testing to include prenatal exposures and how they may link to adult disease. It is clear that there are multiple windows of susceptibility and that exposures early in life may have long-lasting consequences to both health and disease.

Finally, the antiquated idea that the dose makes the poison is overly simplistic. The newest research clearly shows that biology is affected by low doses of chemicals, often within the range of general population exposure, and that these biological changes can be harmful, especially during periods of development. Therefore, low-dose research must go hand in hand with our life-span approach.

The NIEHS’s job doesn’t stop with the publication of scientific results. We also have an obligation to help translate the nation’s research investment into public health intervention, new policy, and preventive clinical practice.

To be successful, we at the NIEHS need to conduct and support the best science, whether it is led by an individual researcher or a multidisiplinary team, and this means working together with all of our partners. We thank everyone who joined us in our strategic planning process. We appreciate your commitment and support.
